# Tanshinone ⅡA Inhibits Alveolar Macrophage Polarization, Inflammation and Mitochondrial Damage by Regulating the PAPR-1 Signaling Pathway

**DOI:** 10.5812/ijpr-166272

**Published:** 2025-11-15

**Authors:** Na Zhang, Xinjia Yang, Jiefei Liang, Chunyan Zhu, Guohua Shen, Chao Luo, Weibin Wu

**Affiliations:** 1 Shaoxing Key Laboratory of Targeted Drug Delivery and Targeted Materials, Department of Medicine and Health, Shaoxing Institute of Technology, Shaoxing, China; 2 School of Basic Medicine, Zhaoqing Medical College, Zhaoqing, China; 3 Affiliated Hospital of Shaoxing University, Shaoxing, China

**Keywords:** Tanshinone IIA, PARP-1, Inflammation, Alveolar Macrophage Polarization, Mitochondrial Damage

## Abstract

**Background:**

Alveolar macrophages (AMs) play a pivotal role in the initiation, resolution, and tissue repair processes of pulmonary inflammatory diseases. The regulation of poly (ADP-ribose) polymerase-1 (PARP-1) is closely associated with inflammatory mechanisms, including the expression of inflammatory mediators, macrophage polarization, and mitochondrial damage. Tanshinone IIA, the primary active component of the Chinese herb *Salvia miltiorrhiza* Bge., exhibits potent anti-inflammatory activity.

**Objectives:**

This study aims to elucidate the mechanism by which tanshinone IIA inhibits macrophage polarization, attenuates the inflammatory response, and prevents mitochondrial damage through regulation of the PARP-1 signaling pathway.

**Methods:**

We investigated the effects of tanshinone IIA on macrophage polarization, inhibition of inflammation and oxidative stress, and protection against mitochondrial damage via the PARP-1 signaling pathway using various experimental approaches, including enzyme-linked immunosorbent assay (ELISA), flow cytometry, western blot analysis, molecular docking, and molecular dynamics (MD) simulation studies.

**Results:**

Compared with the model group, tanshinone IIA significantly inhibited the phosphorylation and activation of nuclear factor kappa B (NF-κB, P < 0.05) in AMs by modulating PARP-1 (P < 0.05). This modulation led to suppression of NLRP3 inflammasome activation (P < 0.05 versus the model group), ultimately inhibiting the release of inflammatory mediators such as nitric oxide (NO), interleukin-1β (IL-1β), interleukin-6 (IL-6), and tumor necrosis factor-alpha (TNF-α, P < 0.05). Simultaneously, tanshinone IIA suppressed cellular oxidative stress via the Nrf2/heme oxygenase-1 (HO-1) pathway (P < 0.05 versus the model group), resulting in decreased reactive oxygen species (ROS) release (P < 0.05) and reduced mitochondrial MitoSOX production (P < 0.05). By regulating PARP-1, tanshinone IIA also effectively inhibited mitochondrial damage (compared with the model group, JC-1 decreased and mitochondrial permeability transition (MPTP) increased, P < 0.05) and M1 polarization of AMs induced by lipopolysaccharide [LPS; expression of CD86, cyclooxygenase-2 (COX-2), and inducible nitric oxide synthase (iNOS) decreased; arginase-1 (ARG-1) and CD206 increased, P < 0.05]. Furthermore, it efficiently modulated signaling pathways involved in mitochondrial fission and fusion [optic atrophy 1 (OPA1), dynamin-related protein 1 (DRP1)] (P < 0.05).

**Conclusions:**

These findings suggest that the therapeutic effects of tanshinone IIA on pulmonary inflammation are closely related to its ability to inhibit inflammatory damage and oxidative stress, regulate AM polarization, and alleviate mitochondrial damage in AMs through modulation of the PARP-1 pathway.

## 1. Background

Poly (ADP-ribose) polymerase-1 (PARP-1) is the most extensively studied member of the PARP family. It is characterized by a zinc-finger DNA-binding domain, an auto-modification domain, and a catalytic domain responsible for adding branched ADP-ribose moieties to target proteins (1). In recent years, numerous studies have shown that PARP-1 is involved in regulating inflammatory damage by modulating the expression of various cytokines, chemokines, and adhesion molecules, thereby playing a key role in the inflammation cycle (2, 3). Our previous studies have also demonstrated that inhibiting PARP-1 expression can significantly alleviate lipopolysaccharide (LPS)-induced acute lung injury (ALI) (4).

The PARP-1 regulates the inflammatory response in various cell types, including innate immune cells (macrophages, neutrophils), adaptive immune cells (lymphocytes), and non-immune cells (such as fibroblasts and endothelial cells) (5, 6). Modulation of PARP-1 expression, alterations in its enzymatic activity, and post-translational modifications can significantly influence the inflammatory activation process of macrophages. In macrophages, PARP-1 plays a crucial role in the regulation of transcription, signaling, inflammasome activity, metabolism, and redox balance. This regulation supports the polarization of macrophages toward the pro-inflammatory M1 phenotype, thereby contributing to excessive inflammatory responses and subsequent tissue damage (7, 8).

Alveolar macrophages (AMs), a critical subset of innate immune cells, are essential for the initiation, resolution, and tissue repair processes associated with lung inflammation (9). Based on activation status, function, and secreted factors, AMs can be categorized into two types: Classically activated M1 macrophages and alternatively activated M2 macrophages. Typically, M1 macrophages are activated by interferon-γ and LPS. They predominantly secrete interleukin-1β (IL-1β), interleukin-6 (IL-6), tumor necrosis factor-alpha (TNF-α), and other inflammatory mediators, which play important roles in the early stages of inflammation (10-12). Furthermore, activated M1 macrophages can recruit monocytes from peripheral blood into the alveoli and promote their polarization into the M1 phenotype, further amplifying the inflammatory response. Therefore, effective regulation of macrophage polarization and inhibition of the subsequent cascade expansion of the inflammatory response are key challenges in the treatment of ALI.

Tanshinone IIA is the principal active ingredient in the Chinese herb *Salvia miltiorrhiza*, renowned for its potent anti-inflammatory activity (13). In the treatment of pulmonary inflammatory diseases, tanshinone IIA has demonstrated significant therapeutic efficacy in conditions such as asthma, ALI, and chronic obstructive pulmonary disease (COPD) (14, 15). It can markedly inhibit inflammatory damage and oxidative stress in lung tissue, reduce pulmonary edema, and suppress the adhesion and migration of neutrophils, macrophages, and other immune cells into the lung tissue. Research has also shown that tanshinone IIA can promote the cleavage of PARP-1 and reduce its expression (16, 17).

## 2. Objectives

Consequently, we hypothesize that the anti-inflammatory activity of tanshinone IIA may be associated with its ability to regulate macrophage polarization, mitigate mitochondrial damage, and modulate the expression of inflammatory factors via the PARP-1 signaling pathway. Therefore, we investigated the regulatory mechanism of tanshinone IIA on macrophage polarization, mitochondrial damage, and inflammatory response using a LPS-induced AM model.

## 3. Methods

### 3.1. Materials and Reagents

Tanshinone IIA was procured from Chengdu Herbpure Biotechnology Co., Ltd. RPMI 1640 medium was purchased from Wuhan Pricella Biotechnology Co., Ltd. β-mercaptoethanol was purchased from Shanghai Macklin Biochemical Technology Co., Ltd. The 0.45 μm PVDF membrane was obtained from Merck Co., Ltd. The TNF-α, IL-1β, and IL-6 enzyme-linked immunosorbent assay (ELISA) kits were procured from Shenzhen Dakewe Biotech Co., Ltd. The NO detection kit, reactive oxygen species (ROS) detection kit, mitochondrial membrane potential detection kit (JC-1), mitochondrial superoxide detection kit (MitoSOX Red), mitochondrial permeability transition pore (MPTP) detection kit, electrophoresis solution, membrane transfer solution, molecular weight standard, BCA protein assay kit, blocking solution, and ECL chemiluminescence kit were purchased from Shanghai Beyotime Biotechnology Co., Ltd. Antibodies for inducible nitric oxide synthase (iNOS), p65, phosphorylated p65 (p-P65), PARP-1, arginase-1 (ARG-1), pro-caspase1, cleaved caspase1, Nrf2, NLRP3, dynamin-related protein 1 (DRP1), heme oxygenase-1 (HO-1), optic atrophy 1 (OPA1), mitofusin 2 (Mfn2), cyclooxygenase-2 (COX-2), CD86, CD206, inhibitor of nuclear factor kappa B-α (IκB-α), phosphorylated inhibitor of nuclear factor kappa B-α (p-IκBα), β-tubulin, and HRP-labeled goat anti-rabbit secondary antibody were purchased from Changzhou Affinity Biosciences Co., Ltd.

### 3.2. Cell Grouping and Drug Treatment

Mouse alveolar macrophages (MH-S) were obtained from Wuhan Pricella Biotechnology Co., Ltd. Cells were maintained in RPMI 1640 medium supplemented with 10% fetal bovine serum (FBS) and 0.05 mM β-mercaptoethanol, and cultured in a humidified incubator at 37°C with 5% CO_2_. Cells were seeded at a density of 1 × 10^6^ cells/mL in 12-well plates and subsequently treated with LPS (10 μg/mL) along with varying concentrations of tanshinone IIA (0, 2.5, 5, and 10 μmol/L) for 24 hours.

### 3.3. Griess Nitrite Assay

The production of NO was assessed using the Griess assay (18). According to the manufacturer’s instructions, 50 μL each of Griess reagent I and II were added to 50 μL of MH-S culture supernatant in a microplate and incubated at room temperature. After 10 minutes, the optical density was measured at 540 nm using a microplate reader (Spark^®^, TECAN, Switzerland), and the concentration of nitrite was determined using sodium nitrite as a standard.

### 3.4. Cytokine Measurement Using Enzyme-Linked Immunosorbent Assay

The concentrations of TNF-α, IL-1β, and IL-6 in cell culture supernatants were measured using ELISA kits according to the manufacturer’s instructions. The absorbance was read at 450 nm on a microplate reader (Spark^®^, TECAN, Switzerland).

### 3.5. Measurement of Reactive Oxygen Species, Mitochondrial Superoxide, Mitochondrial Membrane Potential, and Mitochondrial Permeability

Cell seeding and treatment were performed as described above. After 24 hours of treatment, intracellular ROS, mitochondrial superoxide, mitochondrial membrane potential, and mitochondrial permeability were detected using DCFH-DA, MitoSOX Red, JC-1, and MPTP kits, respectively, in accordance with the manufacturers’ protocols. Flow cytometry was performed using a CytoFLEX (Beckman Coulter, USA).

### 3.6. Western Blot Analysis

Western blot analysis was performed as previously described in our research (19). Total protein was extracted from MH-S cells using RIPA protein lysis buffer, and protein concentrations were determined using a BCA protein assay kit. Equal amounts of protein were separated by 10% SDS-polyacrylamide gel electrophoresis and transferred onto an Immobilon-E PVDF membrane. Membranes were blocked with 5% non-fat milk for 1 hour at room temperature, followed by overnight incubation at 4°C with primary antibodies (1:1000 dilution). After washing, membranes were incubated with an HRP-conjugated secondary antibody (1:5000 dilution). Protein bands were visualized using the ECL detection system, and band intensities were quantified using ImageJ software.

### 3.7. Molecular Docking

The three-dimensional molecular structure of tanshinone IIA was retrieved from the PubChem database (PubChem CID 164676), downloaded and saved in .sdf format. The three-dimensional structure of PARP-1 was obtained from the RCSB Protein Data Bank (PDB ID: 5DS3) and saved in .PDB format. Molecular docking was performed using Glide software (Version 12.0.1, Schrödinger, 2024).

### 3.8. Molecular Dynamics Simulation Studies

Molecular dynamics (MD) simulations were performed using Gromacs 2022. Tanshinone IIA was parameterized with the GAFF force field, while PARP-1 was described by the AMBER14SB force field and the TIP3P water model. The PARP-1 and tanshinone IIA complex was assembled for simulation. Hydrogen bonds were constrained using the LINCS algorithm with a 2 fs integration step. Electrostatic interactions were calculated using the Particle-Mesh Ewald (PME) method with a cutoff of 1.2 nm. Non-bonded interactions used a 10 Å cutoff, updated every 10 steps. The system temperature was maintained at 298 K using the V-rescale method, and the pressure was controlled at 1 bar using the Berendsen method. A 100 ps NVT and NPT equilibration simulation was performed at 298 K, followed by a 100 ns MD production run. Snapshots were saved every 10 ps. Trajectory analyses were performed with VMD and PyMol, and the binding free energy between PARP-1 and tanshinone IIA was evaluated using the g_mmpbsa program.

### 3.9. Statistical Analysis

Data are presented as mean ± standard deviation (SD). Statistical analysis was performed using SPSS 18.0 software. One-way analysis of variance (ANOVA) and the least significant difference (LSD) test were used for data analysis. A P-value less than 0.05 was considered statistically significant.

## 4. Results

### 4.1. Tanshinone IIA Inhibits the Inflammatory Response of Lipopolysaccharide-Induced Alveolar Macrophages

**Figure 1.**
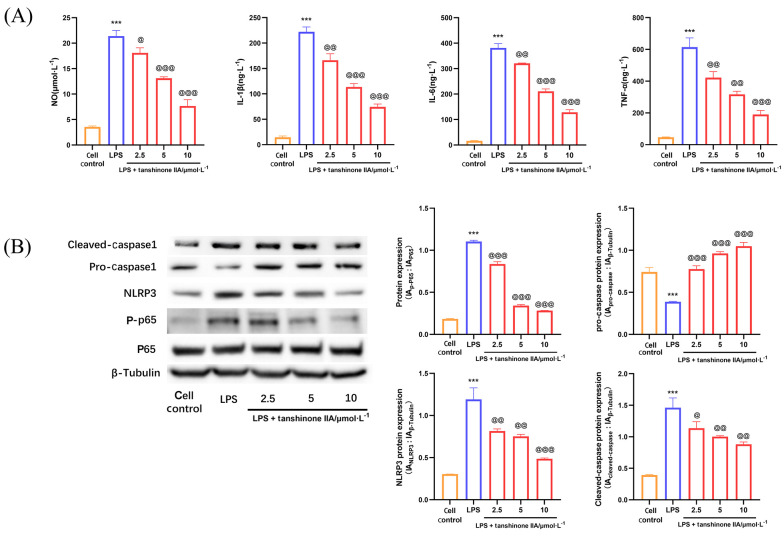
Tanshinone IIA inhibits the inflammatory response of alveolar macrophages (AMs) induced by lipopolysaccharide (LPS): A, pro-inflammatory cytokine levels [nitric oxide (NO), tumor necrosis factor-alpha (TNF-α), interleukin-1β (IL-1β), interleukin-6 (IL-6)] in LPS-induced AMs; B, expression of p65, phosphorylated p65 (p-p65), NLRP3, pro-Caspase1, and cleaved Caspase1 in LPS-induced AMs [n = 3; values are presented as mean ± standard deviation (SD); *** P < 0.001 vs. the control group, @ P < 0.05, @@ P < 0.01, and @@@ P < 0.001 vs. LPS-treated group].

Tanshinone IIA is well known for its superior anti-inflammatory activity. Our experiments demonstrated that tanshinone IIA exerts a significant anti-inflammatory effect on LPS-induced AMs. As shown in Figure 1A, tanshinone IIA (2.5, 5, and 10 μmol × L^-1^) significantly inhibited the expression of pro-inflammatory mediators — nitric oxide (NO), IL-1β, IL-6, and TNF-α — in AMs stimulated by LPS. To further elucidate the molecular mechanisms underlying the protective effect of tanshinone IIA on AMs exposed to LPS, we assessed the expression patterns of nuclear factor kappa B (NF-κB) and NLRP3 proteins in LPS-induced AMs. The results showed that tanshinone IIA has the capacity to inhibit the phosphorylation and activation of NF-κB, thereby suppressing the expression of the NLRP3 inflammasome and inhibiting the cleavage and activation of caspase-1 (Figure 1B). 

### 4.2. Tanshinone IIA Inhibits the Oxidative Damage of Lipopolysaccharide-Induced Alveolar Macrophages

**Figure 2.**
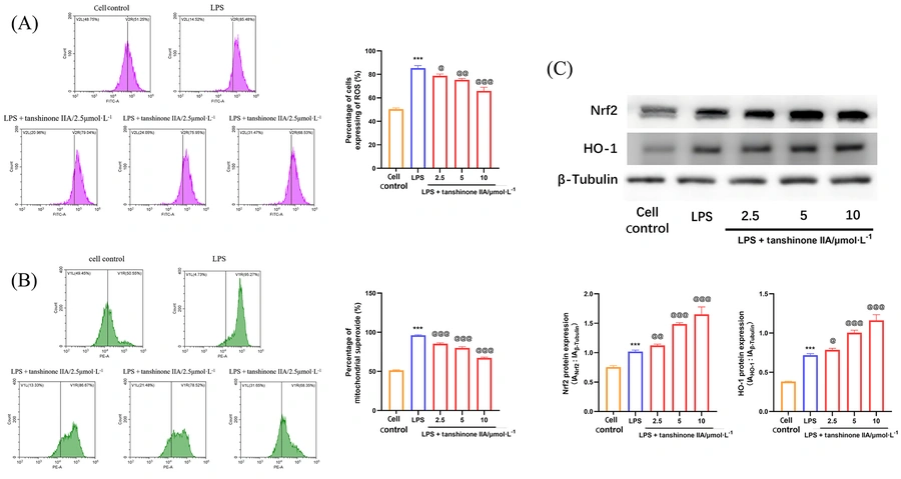
Tanshinone IIA inhibits the oxidative damage of alveolar macrophages (AMs) induced by lipopolysaccharide (LPS): A, intracellular reactive oxygen species (ROS) levels; B, mitochondrial MitoSOX content in LPS-induced AMs; C, expression of Nrf2 and heme oxygenase-1 (HO-1) in LPS-induced AMs [n = 3; values are presented as mean ± standard deviation (SD); *** P < 0.001 vs. the control group, @ P < 0.05, @@ P < 0.01, and @@@ P < 0.001 vs. LPS-treated group].

Oxidative damage frequently accompanies inflammation and can further amplify the inflammatory response. We further investigated the protective effects of tanshinone IIA on LPS-induced oxidative damage in AMs. The study demonstrated that tanshinone IIA (2.5, 5, and 10 μmol × L^-1^) exhibited a marked protective effect against LPS-induced oxidative damage in AMs, significantly suppressing both intracellular ROS levels and mitochondrial superoxide generation (Figure 2A and B). Western blot analysis further revealed that tanshinone IIA modulates the Nrf2/HO-1 pathway, leading to significant upregulation of Nrf2 and HO-1 expression in LPS-induced AMs (Figure 2C). 

### 4.3. Tanshinone IIA Inhibits the Mitochondrial Damage of Lipopolysaccharide-Induced Alveolar Macrophages

**Figure 3.**
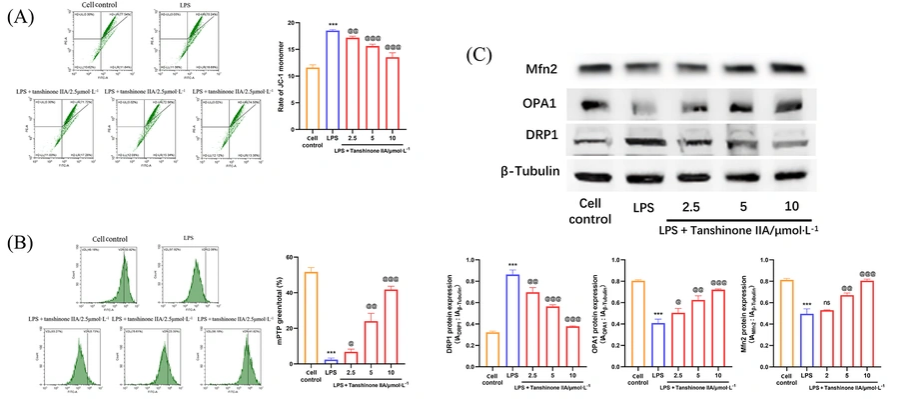
Tanshinone IIA inhibits the mitochondrial damage of alveolar macrophages (AMs) induced by lipopolysaccharide (LPS): A, mitochondrial membrane potential levels; B, mitochondrial permeability in LPS-induced AMs; C, expression of dynamin-related protein 1 (DRP1), optic atrophy 1 (OPA1), and mitofusin 2 (Mfn2) in LPS-induced AMs [n = 3; values are presented as mean ± standard deviation (SD); *** P < 0.001 vs. the control group, @ P < 0.05, @@ P < 0.01, and @@@ P < 0.001 vs. LPS-treated group].

Mitochondria play a pivotal role in cellular energy metabolism, and mitochondrial damage significantly promotes inflammation and oxidative stress (20). We further evaluated the mitochondrial protective effects and underlying mechanisms of tanshinone IIA on macrophages subjected to inflammatory injury. The results indicated that tanshinone IIA can markedly alleviate mitochondrial damage induced by LPS in AMs. Mitochondrial membrane potential and mitochondrial permeability assays revealed that the proportion of JC-1 monomer was significantly increased in the LPS-induced group (Figure 3A), and green fluorescence of MPTP was quenched (Figure 3B), suggesting compromised mitochondrial activity, decreased membrane potential, and increased permeability. In contrast, the tanshinone IIA treatment groups (2.5, 5, and 10 μmol × L^-1^) exhibited a significant reduction in the proportion of JC-1 monomer and a notable enhancement in green fluorescence intensity of MPTP. Western blot results showed that tanshinone IIA inhibited DRP1 translocation and increased the expression of fusion proteins OPA1 and Mfn2, thereby suppressing mitochondrial fission and maintaining mitochondrial structural stability (Figure 3C). 

### 4.4. Tanshinone IIA Regulates the M1/M2 Polarization of Lipopolysaccharide-Induced Alveolar Macrophages

**Figure 4.**
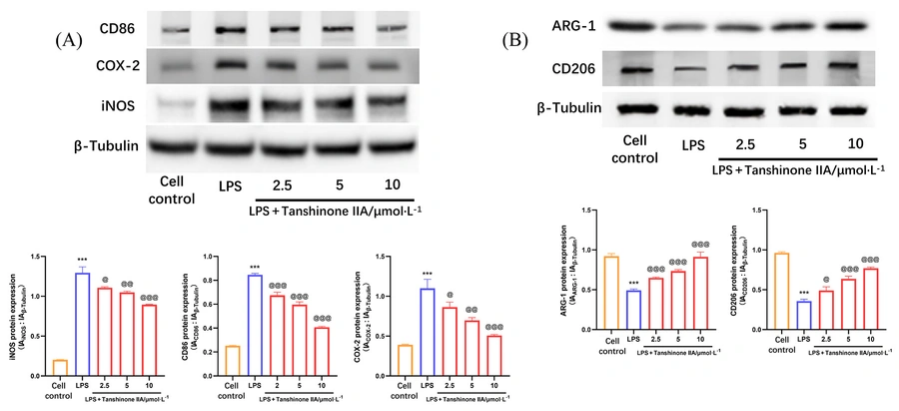
Tanshinone IIA regulates the M1/M2 polarization of alveolar macrophages (AMs) induced by lipopolysaccharide (LPS): A, expression of M1 polarization markers [CD86, cyclooxygenase-2 (COX-2), inducible nitric oxide synthase (iNOS)] in LPS-induced AMs; B, expression of M2 polarization markers [CD206, arginase-1 (ARG-1)] in LPS-induced AMs [n = 3; values are presented as mean ± standard deviation (SD); *** P < 0.001 vs. the control group, @ P < 0.05, @@ P < 0.01, and @@@ P < 0.001 vs. LPS-treated group].

Studies have demonstrated that LPS stimulation promotes M1 polarization in AMs, triggering an inflammatory response (21). Our experimental results corroborated that LPS induces increased expression of M1 polarization markers such as iNOS, CD86, and COX-2 in AMs, while decreasing the expression of M2 polarization markers including ARG-1 and CD206 (Figure 4). Tanshinone IIA treatment effectively suppressed the M1 polarization induced by LPS in AMs. Compared with the LPS group, the tanshinone IIA treatment groups (2.5, 5, and 10 μmol × L^-1^) demonstrated a significant reduction in iNOS, CD86, and COX-2 expression, alongside a significant increase in ARG-1 and CD206 expression.

### 4.5. Tanshinone IIA Regulates the Poly (ADP-ribose) Polymerase-1 Signaling Pathway in Lipopolysaccharide-Induced Alveolar Macrophages

**Figure 5.**
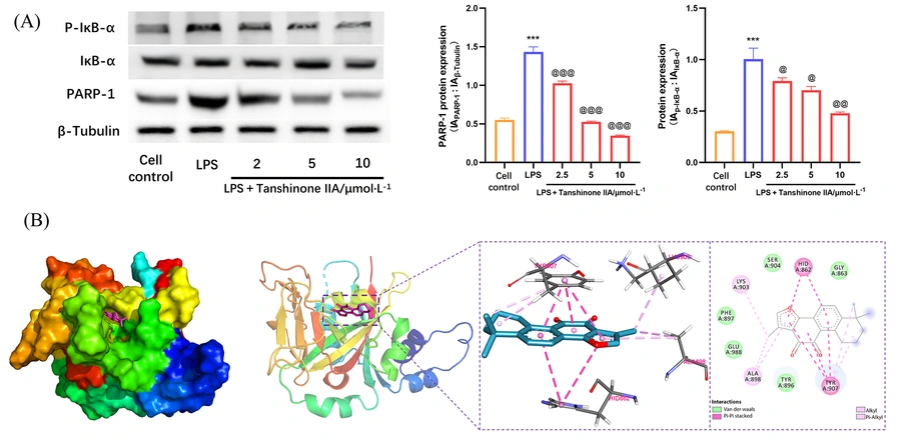
Tanshinone IIA regulates the poly (ADP-ribose) polymerase-1 (PARP-1) pathway in alveolar macrophages (AMs) induced by lipopolysaccharide (LPS): A, expression of PARP-1, inhibitor of kappa B-α (IκB-α), and phosphorylated inhibitor of kappa B-α (p-IκB-α) in LPS-induced AMs; B, schematic diagram of molecular docking of tanshinone IIA with PARP-1 [n = 3; values are presented as mean ± standard deviation (SD); *** P < 0.001 vs. the control group, @ P < 0.05, @@ P < 0.01, and @@@ P < 0.001 vs. LPS-treated group].

**Figure 6.**
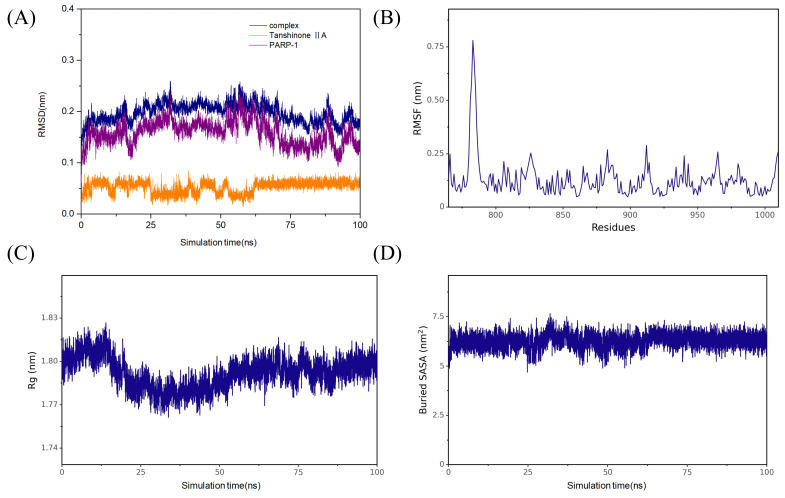
Molecular dynamics (MD) simulation results of tanshinone IIA and poly (ADP-ribose) polymerase-1 (PARP-1): A, root mean square deviation (RMSD) of the protein backbone, B, root mean square fluctuation (RMSF) of amino acid residues in the protein; C, radius of gyration of the protein backbone; and D, solvent-accessible surface area of the protein.

The PARP-1 signaling pathway is closely linked to inflammation, oxidative damage, and macrophage polarization, among other processes (22, 23). Our molecular docking studies showed that tanshinone IIA can effectively interact with PARP-1 (Figure 5B). Within PARP-1, amino acids His-862, Tyr-907, Ala-898, and Lys-903 formed hydrophobic interactions with tanshinone IIA via Pi-Pi stacking, alkyl, and Pi-alkyl mechanisms. In addition, amino acids such as Ser-904, Phe-897, and Tyr-896 established van der Waals interactions with tanshinone IIA. Western blot analysis demonstrated that tanshinone IIA significantly inhibits PARP-1 and suppresses activation of the PARP-1 signaling pathway, reducing the phosphorylation of downstream signaling molecule IκB-α (Figure 5A). Further MD simulation results indicated that tanshinone IIA binds stably to PARP-1. Analysis of binding energy revealed that van der Waals interactions are the primary determinant, followed by hydrophobic interactions, with electrostatic interactions contributing as a tertiary factor (Figure 6). 

## 5. Discussion

The AMs, a major component of the innate immune system, reside in the alveoli and perform critical functions including phagocytosis, both pro-inflammatory and anti-inflammatory activities, and the activation of T helper 1 (Th1) and T helper 2 (Th2) responses (24). The AMs are essential for maintaining pulmonary immune homeostasis, and disturbances in their inflammatory response, oxidative balance, and polarization status contribute significantly to the onset and progression of acute pulmonary inflammatory diseases such as ALI. It is well established that LPS induction promotes M1 polarization in AMs, subsequently stimulating the secretion of pro-inflammatory mediators such as IL-1β, IL-6, TNF-α (12). The release of these pro-inflammatory factors further amplifies inflammation, leading to increased lung damage. Moreover, excessive inflammation reciprocally promotes oxidative and mitochondrial damage.

The PARP-1 is implicated in regulating numerous inflammatory processes (6), closely associated with the activation of immune cells — including macrophages, neutrophils, dendritic cells, and lymphocytes — as well as the inflammatory responses of non-immune cells such as fibroblasts and endothelial cells. Among these, the polarization and activation of AMs play a pivotal role in the acute phase of pulmonary inflammation (11). Research has shown that PARP-1 is crucial in regulating LPS-induced macrophage polarization, enhancing polarization towards the M1 phenotype by affecting both transcriptional and post-transcriptional processes. The PARP-1 facilitates the expression and mRNA stability of pro-inflammatory factors. In response to LPS, PARP-1 is phosphorylated at the conserved Y829 site by the c-Abl tyrosine kinase, which in turn activates NF-κB (7). The NF-κB is a key transcription factor governing the expression of inflammation-related genes and orchestrating innate immune responses. The activation of NF-κB in macrophages initiates an inflammatory cascade and promotes their recruitment to sites of infection, where activated M1 macrophages secrete TNF-α, IL-1β, IL-6, and other cytokines to mobilize neutrophils and initiate the host response (25).

The activity of NF-κB is regulated in part by PARP-1. The inhibitor of kappa B (IκB) retains NF-κB in the cytoplasm in resting cells, preventing its nuclear localization and transcriptional activity (26). The PARP-1 activates and phosphorylates inhibitor of kappa B kinase (IKK), which phosphorylates IκB, promoting its degradation and the release of NF-κB, thereby enabling its nuclear translocation and activation. The p65 subunit of NF-κB (p-p65) is phosphorylated and translocates to the nucleus to drive transcription of target genes (27).

Tanshinone IIA is widely recognized for its potent anti-inflammatory properties and exhibits significant therapeutic efficacy in various pulmonary inflammatory diseases, including ALI (28). Previous studies have explored the mechanisms by which tanshinone IIA treats pulmonary inflammation, demonstrating its ability to inhibit NF-κB (29), suppress NLRP3 inflammasome activation (30), and regulate macrophage polarization (15). However, the regulatory mechanism of tanshinone IIA on pulmonary inflammation-related signaling pathways via PARP-1 has not been systematically studied.

Our findings demonstrate that the anti-inflammatory effects of tanshinone IIA are mediated by its suppression of inflammatory factor expression and M1 polarization of AMs through the PARP-1 pathway. Molecular docking and molecular dynamics simulation studies revealed a strong binding affinity of tanshinone IIA to PARP-1. Western blot analysis further confirmed that tanshinone IIA inhibits PARP-1 and its associated signaling pathway, resulting in the suppression of IκB-α phosphorylation. This, in turn, inhibits the phosphorylation and activation of NF-κB p65, suppresses NLRP3 inflammasome activation, and ultimately reduces the expression of pro-inflammatory factors.

Mitochondria are critical organelles involved in cellular energy metabolism, signal transduction, and apoptosis. Studies have shown that mitochondrial damage and cellular inflammatory responses are mutually reinforcing (31). Inflammatory responses can induce mitochondrial damage, and inflammatory cytokines can significantly alter mitochondrial dynamics and compromise mitochondrial energy metabolism, ultimately leading to apoptosis (32). Damaged mitochondria can further amplify inflammation by overproducing mitochondrial ROS and releasing damage-associated molecular patterns (DAMPs) (33). Additionally, alterations in mitochondrial energy metabolism can influence macrophage polarization (34).

The PARP-1 hyperactivity can indirectly affect metabolic homeostasis. Excessive activation of PARP-1 leads to depletion of nicotinamide adenine dinucleotide (NAD^+^), resulting in reduced glycolysis, impaired electron transport chain function, and decreased adenosine triphosphate (ATP) production — ultimately compromising mitochondrial and cellular function (35). Furthermore, PARP-1 is involved in the regulation of mitochondrial fission and fusion protein expression, and its inhibition can enhance mitochondrial stability (36, 37). Our results indicate that tanshinone IIA may directly prevent mitochondrial damage caused by NAD^+^ depletion through the inhibition of PARP-1 and may also regulate mitochondrial fission/fusion balance by inhibiting PARP-1. Additionally, tanshinone IIA reduces cellular inflammatory injury via the PARP-1 pathway, thereby mitigating mitochondrial damage.

The development of therapeutics targeting the PARP-1 pathway for regulation of inflammatory responses is gaining increasing attention. Our study supports the hypothesis that the therapeutic effects of tanshinone IIA in pulmonary inflammatory diseases are attributable to its interaction with the PARP-1 signaling pathway, a finding with significant implications for the future application and exploration of tanshinone IIA. Nevertheless, our research is currently limited to in vitro mechanistic studies. Further in vivo investigations and comprehensive mechanistic analyses will be required to validate these findings.

## Data Availability

The data are available from the corresponding author upon reasonable request.
